# Murine models of renal ischemia reperfusion injury: An opportunity for refinement using noninvasive monitoring methods

**DOI:** 10.14814/phy2.15211

**Published:** 2022-03-09

**Authors:** Rachel Harwood, Joshua Bridge, Lorenzo Ressel, Lauren Scarfe, Jack Sharkey, Gabriela Czanner, Philip A Kalra, Aghogho Odudu, Simon Kenny, Bettina Wilm, Patricia Murray

**Affiliations:** ^1^ Institute of Translational Medicine University of Liverpool Liverpool UK; ^2^ Alder Hey Children's Hospital Liverpool UK; ^3^ Department of Biostatistics University of Liverpool Liverpool UK; ^4^ Department of Eye and Vision Science University of Liverpool Liverpool UK; ^5^ Department of Veterinary Pathology and Public Health University of Liverpool Liverpool UK; ^6^ University of Liverpool Liverpool UK; ^7^ University of Liverpool Liverpool UK; ^8^ Division of Cardiovascular Sciences University of Manchester Manchester UK; ^9^ Salford Royal NHS Foundation Trust Salford UK

**Keywords:** acute kidney injury, chronic kidney disease, ischemia reperfusion injury

## Abstract

**Background:**

Renal ischemia reperfusion injury (R‐IRI) can cause acute kidney injury (AKI) and chronic kidney disease (CKD), resulting in significant morbidity and mortality. To understand the underlying mechanisms, reproducible small‐animal models of AKI and CKD are needed. We describe how innovative technologies for measuring kidney function noninvasively in small rodents allow successful refinement of the R‐IRI models, and offer the unique opportunity to monitor longitudinally in individual animals the transition from AKI to CKD.

**Methods:**

Male BALB/c mice underwent bilateral renal pedicle clamping (AKI) or unilateral renal pedicle clamping with delayed contralateral nephrectomy (CKD) under isoflurane anesthetic. Transdermal GFR monitoring and multispectral optoacoustic tomography (MSOT) in combination with statistical analysis were used to identify and standardize variables within these models.

**Results:**

Pre‐clamping anesthetic time was one of the most important predictors of AKI severity after R‐IRI. Standardizing pre‐clamping time resulted in a more predictably severe AKI model. In the CKD model, MSOT demonstrated initial improvement in renal function, followed by significant progressive reduction in function between weeks 2 and 4. Performing contralateral nephrectomy on day 14 enabled the development of CKD with minimal mortality.

**Conclusions:**

Noninvasive monitoring of global and individual renal function after R‐IRI is feasible and reproducible. These techniques can facilitate refinement of kidney injury models and enable the degree of injury seen in preclinical models to be translated to those seen in the clinical setting. Thus, future therapies can be tested in a clinically relevant, noninvasive manner.

## Graphical abstract

Noninvasive methods of measuring murine glomerular filtration rate (GFR) are used to refine both acute and chronic models of renal ischemia reperfusion injury (r‐IRI). Transdermal GFR monitoring revealed that standardization of the anesthetic duration prior to clamping reduces the variability of the acute injury model. Multispectral optoacoustic tomography (MSOT) enabled refinement of the chronic kidney disease model resulting in sustained reduction of the global GFR after r‐IRI in a humane way.

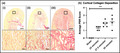

## 1 INTRODUCTION

Acute kidney injury (AKI) and chronic kidney disease (CKD) are leading causes of morbidity and mortality (Susantitaphong et al., 2013). Reduced renal perfusion causing AKI can be attributed to sepsis, hypovolaemia (Rahman et al., 2012), cardiac bypass surgery (Chien et al., 2009), and renal pedicle clamping during partial nephrectomy (Bessede et al., 2015). These lead to renal ischemia‐reperfusion injury (R‐IRI) which progresses from AKI to functional recovery in most and CKD in some (Rahman et al., 2012). Childhood AKI significantly increases the risk of developing CKD (Morgan et al., 2015; Newnham & Friend, 2018) and AKI in an adult with CKD accelerates their progression toward end‐stage renal disease (ESRD) (Ishani et al., 2009).

Therapeutic adjuncts are needed to reduce the severity of AKI and progression to CKD, but therapies are lacking. A range of potential treatments have been investigated, but none have shown efficacy in clinical trials (BMJ, 2017). In view of this ongoing need, robust preclinical evaluation of novel therapies for AKI and CKD is required.

R‐IRI is a preclinical model of AKI (Ortiz et al., 2015) caused by unilateral or bilateral clamping of renal pedicles, and in the former, with or without contralateral nephrectomy (CN). Creation of R‐IRI varies between groups who use differing anesthetic agents, clamping times, temperatures, and approaches to the kidney (Hesketh et al., 2014; Skrypnyk et al., 2013; Wei & Dong, 2012), all of which alter the severity of R‐IRI AKI. Additionally, the genetic background and strain of animal can affect the AKI to CKD progression (Scarfe et al., 2019). Because R‐IRI is a notoriously variable model of kidney injury (Wei & Dong, 2012), its refinement enables fewer animals to be used when assessing therapeutic effects.

Bilateral R‐IRI creates an injury affecting global glomerular filtration rate (GFR) and is classically measured using blood markers of kidney function (Diskin, 2006; Hosten, 1990). In small rodents these are typically performed at the experimental endpoint due to the volumes of blood required to undertake analysis of multiple serum markers of injury (Scarfe et al., 2015). Creatinine, the most commonly used marker of injury has a nonlinear relationship with GFR, meaning that a substantial reduction in GFR is required before an increase in creatinine is seen (Pasala & Carmody, 2017). Creatinine can be measured using small volumes of serum and is classically measured using a modified Jaffe reaction. This can be impacted on by hemolysis (Nigam, 2016), making a panel of serum markers necessary to assess renal function and meaning that longitudinal measurements are often not possible.

By contrast, noninvasive methods of measuring renal clearance enable longitudinal monitoring of kidney function in the same animal (Herrera Perez et al., 2016; Roberts et al., 2007; Schreiber et al., 2012). Transdermal measurement of FITC‐Sinistrin clearance time allows calculation of global GFR (Scarfe et al., 2018; Schreiber et al., 2012), making it an ideal tool for monitoring renal function after R‐IRI and reducing animal numbers.

The kidney disease improving global outcomes (KDIGO) group released guidance in 2012 (Palevsky et al., 2013) amalgamating previous AKI stratification tools; acute dialysis quality initiative (ADQI) (Bellomo et al., 2004) who defined the “RIFLE” classification and acute kidney injury network (AKIN) (Mehta et al., 2007) guidance. KDIGO use serum creatinine and urine output to define AKI which, as described above, cannot be used longitudinally to monitor AKI in small rodents. However, the percentage reduction in rodent GFR can be compared to the ADQI classification using “Risk” (>25% GFR decrease), “Injury” (>50% GFR decrease), “Failure” (>75% GFR decrease), and “Loss” (complete loss of function) (Bellomo et al., 2004) using transdermal GFR monitoring. Humans in the “Failure” group have a 46.5% mortality rate (Hoste et al., 2006), so applying this clinically relevant preclinical level of AKI enables therapies to be trialed appropriately (Denayer et al., 2014).

Unilateral R‐IRI with delayed contralateral nephrectomy (CN) enables severe AKI to be induced because the healthy kidney provides function while the severely injured kidney recovers and enables assessment of severe AKI and progression to CKD. When unilateral R‐IRI is performed without CN the overall GFR does not reflect the injured kidney's function, which will be substantially less than the uninjured kidney. This makes establishing the optimal time point for removing the healthy kidney to allow transdermal GFR monitoring of the injured kidney difficult. From a National Centre for the Replacement Refinement & Reduction of Animals in Research (NC3R) perspective, refinement of this model is important because if the healthy kidney is removed before the injured kidney has regained sufficient function, the animal will suffer.

IRDye 800 clearance measured using multispectral optoacoustic tomography (MSOT) correlates well with histologically quantified kidney damage in an Adriamycin model of injury (Huang & Gretz, 2017; Scarfe et al., 2015). A recent study has shown that IRDye 800 clearance can be measured repeatedly and reproducibly in the kidneys of healthy mice using MSOT, indicating that longitudinal studies could be performed with high confidence (Joseph et al., 2017). In unilateral R‐IRI, noninvasive monitoring of both injured and healthy kidney using MSOT could enable the function of each kidney be simultaneously tracked.

We aimed to refine acute and chronic ischemic models of kidney injury using noninvasive methods of monitoring kidney function. We used a validated transdermal device to measure GFR longitudinally in mice, in order to establish the effect of clamping time on GFR after bilateral R‐IRI (Scarfe et al., 2015, 2019). This allowed us to determine whether GFR returned to baseline levels after 2 weeks. It also enabled us to explore whether the GFR impairment occurring immediately after R‐IRI can predict the degree of subsequent histological damage. By assessing the contribution of anesthetic time to injury severity we refined the model to reduce variability. Finally, we developed clinically relevant models of AKI, including severe AKI leading to CKD, while remaining NC3R compliant. To this end, we used an emerging technology, MSOT, which enables noninvasive visualization of the kidneys, and longitudinal monitoring of function.

## 2 MATERIALS AND METHODS

### 2.1 Animals

All experimentation was performed under a Project Licence (PPL 7008741) granted by the Home Office under the Animals (Scientific Procedures) Act 1986. Male BALB/c mice (Charles River) aged 8–10 weeks were used. Mice were housed in individually ventilated cages in a 12‐hour light/dark cycle. They had ad libitum access to food and water and were acclimatized for 1 week before the study. Experiments are reported in line with the ARRIVE guidelines (see Supplementary Information 1.e).

### 2.2 AKI model

Briefly, after induction of anesthesia with inhaled oxygenated isoflurane, the animal was transferred to a heat pad with a rectal temperature probe and a feedback‐regulated system, body temperature set to 37°C. Surgery was commenced after the temperature reached 36.5°C. A dorsal approach was taken (Skrypnyk N. et al., 2013) and an atraumatic vascular clamp (Schwartz, Interfocus, Linton) was placed on the vessels. Clamp times of 25, 27.5, and 30 min were used with six animals per group (Supplementary Information 1a and 1.b). These clamp times were used as large differences between clamp times have been investigated and reported on, but the fine‐tuning of the r‐IRI model to produce a severe but survivable AKI that is representative of patients who require therapy the most has not been thoroughly reported. The duration of anesthesia before application of the first clamp and the time between removal of the second clamp and the mouse entering the postoperative warmed chamber (post‐clamp time) was recorded. Three mice underwent sham operations. Transdermal FITC‐Sinistrin clearance was measured preoperatively and days 1, 3, 7, and 14 after surgery. After monitoring the acute temporal changes in GFR, animals were culled on day 14 and blood and kidneys collected.

### 2.3 Refined AKI model

A refined AKI model was performed in six animals. The presurgical anesthetic time was standardized to 30 min between the start of anesthetic and commencement of surgery (see Supplementary Information 1.c and Figure S1). Transdermal GFR was measured preoperatively and days 1 and 3 postoperatively. Animals were culled on day 3 and blood and kidneys were collected.

### 2.4 CKD model

To produce R‐IRI‐induced CKD a pilot study was performed with prolonged unilateral renal pedicle clamping (40 min) in three mice using the “Refined AKI model” of the left renal pedicle. MSOT was undertaken on multiple days after injury, and nephrectomy of the uninjured kidney was performed on day 29. GFR was measured on day 30, animals were culled, and blood and kidneys retrieved (Figure S2a).

### 2.5 Refined CKD model

The model was refined by performing right‐sided renal pedicle clamping in six animals, enabling better visualization of the kidney with MSOT. The CN was performed on day 14 and GFR measurements taken from day 21 to the endpoint of the experiment, 6 weeks after R‐IRI (Figure S2b). Early GFR measurements after contralateral nephrectomy were not undertaken as the intention was to produce a stable CKD model. Early GFR measurement after nephrectomy may cause additional unnecessary stress to the animal and reflect physiological changes such as reduced fluid intake as well as intrinsic renal function. These changes cannot be avoided in the AKI model but are not the focus of the CKD model.

### 2.6 Transdermal FITC‐Sinistrin clearance measurement

FITC‐Sinistrin clearance was measured in all mice, as previously described (Scarfe et al., 2018). The half‐life of FITC‐Sinistrin was converted to GFR using the formula (Schreiber et al., 2012).

$$\begin{array}{c} \text{GFR}\left[\mu l/\min/100\;g\;\text{bw}\right]=\\ 14616.8\left[\mu l/100\;g\;\text{bw}\right]/t_{1/2}\left(\text{FITC ‐ Sinistrin}\right)\left[\min\right]. \end{array}$$

enabling the GFR of mice with no excretion to be included in the analysis.

Absolute and proportional GFRs were compared to baseline measurements. The proportional change was correlated with the “RIFLE” classification of kidney injury, depicting the levels of risk, injury, and failure as described by the ADQI working group (Bellomo et al., 2004). The values for each level were calculated from the mean of the baseline GFR measurements, which followed a normal distribution (Figure S3).

### 2.7 Multispectral optoacoustic tomography (MSOT)

The inVision 256‐TF MSOT imaging system (iThera Medical) was employed in the CKD model, using IRDye 800 to measure renal kinetics in the injured and healthy contralateral kidneys (details of acquisition and analysis within Supplementary Information 1.d).

#### 2.7.1 Serum analysis

Animals were culled in a rising concentration of CO_2_. Once death was confirmed, cardiac puncture was performed to collect blood. This was clotted for 2 h and centrifuged at 2000 g for 20 min. Serum was collected and re‐centrifuged at 75 g for 4 min to remove all blood. Samples were stored at −20°C until analysis, repeat freeze‐thawing was avoided. Creatinine (Detect X Serum Creatinine Detection Kit, Arbor Assays) was assessed using the modified Jaffe Reaction. Urea (QuantiChrom Urea Assay Kit, BioAssay Systems) was assessed using a colorimetric reaction and Cystatin C (Quantikine ELISA, R&D Systems) was analyzed using an ELISA method.

#### 2.7.2 Histopathology

Kidneys were collected and fixed in 4% paraformaldehyde for 1 day. They were processed in paraffin and sections of 4 µm were stained with Picrosirius Red (PSR) to assess for cortical deposition of collagen I and III. Sections were analyzed by a veterinary histopathologist blinded to the intervention. Cortical collagen deposition was graded 0–4 in a single section of each kidney using brightfield light to assess the whole section. Grading was based on the percentage of the cortex judged to show increased collagen deposition by the histopathologist (Figure S6). Blinded grading rather than quantitative analysis was employed as the experienced histopathologist can give an insight into normal and abnormal collagen deposition.

### 2.8 Statistical analysis

Two‐way ANOVA was used to compare GFR at different time points after injury, serum, and histological results. A mixed effects model was used to describe longitudinal weight data of all surviving animals and to analyze t_max_ results as measured using MSOT (time from administration of IRDye800 to the peak of the curve). A post‐analysis Bonferroni correction for comparisons between all groups was applied. Linear regression analysis compared GFR with variables including anesthetic time, heat pad and surgical order, and serum and histological markers of kidney injury. Significance was taken as *p *< 0.05.

## 3 RESULTS

### 3.1 In bilateral R‐IRI, GFR is inversely correlated with subsequent collagen deposition

**FIGURE 1:**
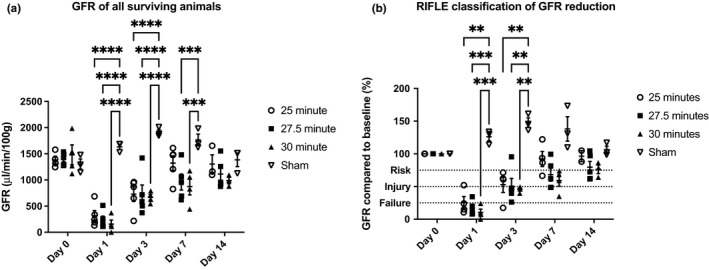
GFR results of animals undergoing bilateral R‐IRI for 25, 27.5 and 30 min and sham procedures. (a) Absolute change in GFR after R‐IRI in animals who survived to the endpoint of the experiment. (b) Proportional change in GFR after bilateral R‐IRI. The ADQI ‘RIFLE’ criteria are shown within the figure. *n* = 6: per clamped group, *n* = 3: sham. All figures show mean ± SEM. **p < 0.01 compared to sham group, ***p < 0.001, ****p < 0.0001 compared to sham group

Bilateral R‐IRI was performed using three different clamp times—25, 27.5, and 30 min—to establish which was best able to induce a severe, survivable injury. Compared with the sham group, the GFR of all injured animals was significantly reduced on day 1, irrespective of clamp time, but had returned to near baseline levels by day 14. There was no significant difference between R‐IRI groups, but the trend was for the GFR to be lower in animals with longer clamp times (Figure 1a and supplementary information). The proportional GFR change showed that the majority of animals entered the RIFLE “failure” category on day 1 after injury in the 27.5 and 30 min groups, but had mostly recovered by day 14 (Figure 1b and Supplementary Information). It was surprising to find that the GFR was not substantially affected by longer clamp times, raising the question of whether other extrinsic factors, such as anesthetic time, could affect the severity of injury.

**FIGURE 2:**
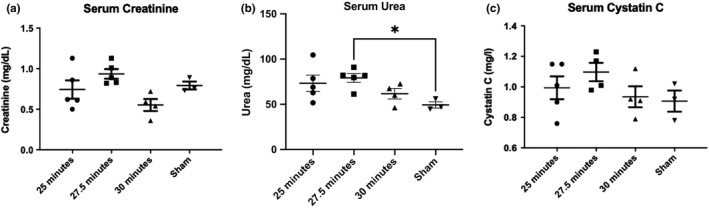
Serum analysis on day 14 after R‐IRI. (a) Serum creatinine, assayed using the modified Jaffe method. (b) Serum Urea. (c) Serum Cystatin (c) There was no significant difference between the treatment groups and the sham group for serum creatinine and cystatin C. The serum urea was higher in the group having a clamping time of 27.5 min compared to sham animals (*p* 0.03)

**FIGURE 3:**
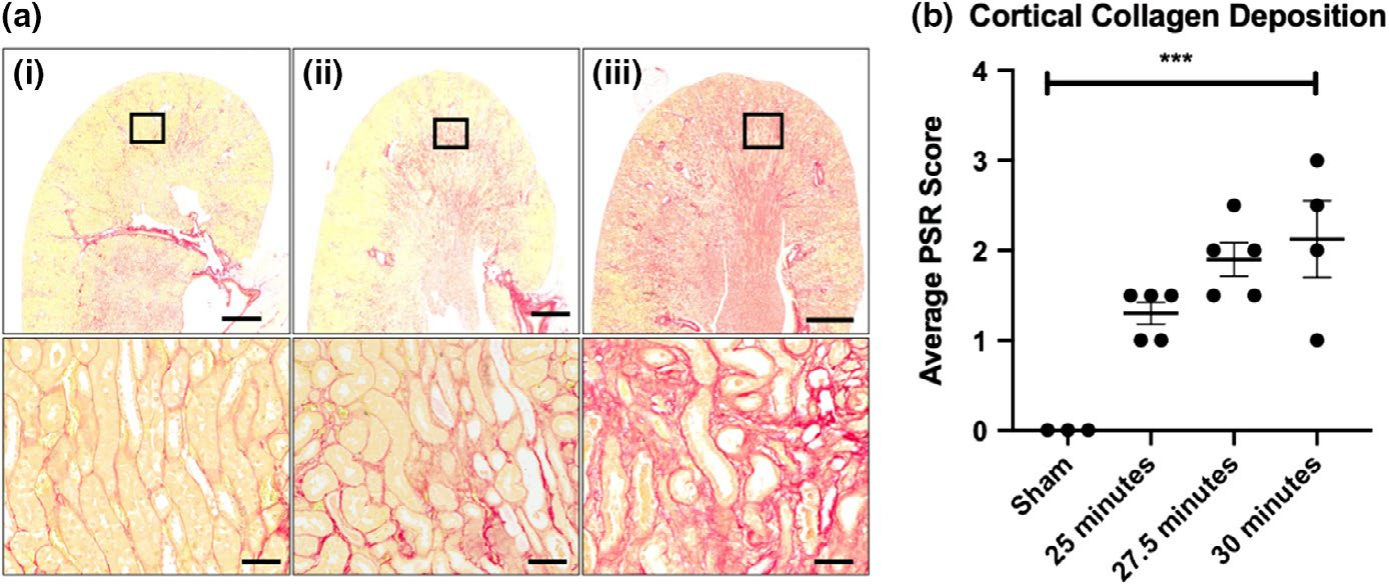
Cortical collagen deposition after R‐IRI on day 14. (a) Representative sections of collagen deposition after each clamp time, lower row zoomed image of box above: (i) 25 min clamp time, (ii) 27.5 min clamp time, (iii) 30 min clamp time. Scale bars: 1 mm upper row, 20 μm lower row. (b) Effect of clamping time on development of collagen deposition (Mean, SEM). Sham‐operated animals had minimal collagen deposition. **p < 0.001 compared to sham group when compared by one‐way ANOVA

There was weight loss in all groups with the nadir occurring 4 days after R‐IRI (Figure S4a). The mortality rate was higher in the 30 min group (33% compared with 17% in the 27.5 and 25 min groups; Figure S4b). At the study endpoint (day 14), no biologically relevant differences in serum creatinine and cystatin‐C were observed compared to sham controls (Figure 2 and supplementary information) but there was evidence of collagen deposition in all injured animals (Figure 3). A clamp‐time of 27.5 min was used in subsequent bilateral R‐IRI experiments because the mortality rate was lower than the 30 min group but there was a strong injury response similar to the 30 min group.

### 3.2 Pre‐clamping anesthetic time correlates with the degree of GFR impairment

The model of bilateral R‐IRI is notoriously variable (Skrypnyk et al., 2013), even when clamp times and temperature are standardized. The total anesthetic times were 78 min (Std Dev 13), 87 min (Std Dev 8), and 85 min (Std Dev 9) for the clamp times of 25, 27.5, and 30 min, respectively. There was no significant difference between the total anesthetic times for each group, showing that a longer clamping time did not equate to a longer anesthetic time (*p* 0.40).

**FIGURE 4:**
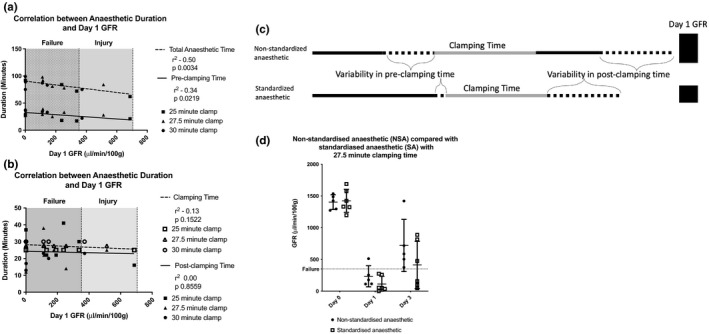
Impact of experimental variables on day 1 GFR measurement which is shown in relation to the ADQI defined category of renal failure (reduction to <25% of the baseline GFR) and the GFR findings after standardisation of the pre‐clamping time to 30 min. (a) Significant correlation of GFR with the total anaesthetic time and pre‐clamping time. (b) No correlation between GFR and clamping time or post‐clamping time. (c) Pictorial representation of the variation in experimental technique. The value of the day 1 GFR is represented by the height of the blue box. (d) Significant reduction in variance of the pre‐clamping anaesthetic time results in a non‐significant reduction in day 1 GFR, with all animals being within the ‘failure’ group

To determine the impact of external experimental variables on GFR, univariate linear regression analysis was performed. This revealed that, independent of clamping time, the total anesthetic time had a significant impact on the day 1 GFR (Figure 4a). It was important to understand if one component of the procedure had more of an impact than others to help to tailor the surgical approach. When considering each constituent of the anesthetic time, the GFR (categorized using the ADQI defined RIFLE criteria) was particularly affected by the time between anesthetic commencement and placement of first clamp (Figure 4a). There was no significant relationship between clamping time or post‐clamping time and GFR (Figure 4b), nor surgical order, which heat pad was used, or the animal's weight.

Based on these observations, we refined the bilateral R‐IRI model (27.5 min clamp time) by standardizing the presurgical anesthetic time to 30 min (start of anesthetic to start of surgery), while continuing to monitor total anesthetic duration. We compared this standardized anesthetic (SA) group to the previously described non‐standardized anesthetic (NSA) group and found that the SA group had a reduction in day 1 GFR (Table 1, Figure 4c,d), suggesting that a more severe but more reproducible injury level was achieved. Standardizing presurgical time meant that surgery was more regimented, resulting in a significantly shorter overall anesthetic time, but a longer, less variable pre‐clamping time. This highlights that it is pre‐clamping anesthetic time, rather than total anesthetic duration, that has the greatest impact on the GFR.

**TABLE 1:** Comparison of non‐standardized and standardized presurgical anesthetic time of animals having a 27.5 min clamp time

	Non‐standardized presurgical anesthetic time	Standardized presurgical anesthetic time
Day 1 GFR (μl/min/100 g)	195 (176.6)	113 (121.3)
Total anesthetic time (min)	86.6 (8.2)	71.7 (9.5)
Pre‐clamping anesthetic time (min)	30.8 (5.4)	34.0 (1.5)

Note

The GFR is reduced to within the “Failure” category (<353µl/min/100g) for all animals who had a standardized presurgery anesthetic time (SA) compared to those who has a non‐standardized anesthetic time (NSA). The pre‐clamping anesthetic time is much more tightly controlled with a significantly smaller variance (p 0.02). Values are Mean (St Dev).

### 3.3 Assessing individual renal function in a mouse model of R‐IRI‐induced CKD using MSOT

A limitation of the bilateral R‐IRI model is that it does not model the most severe AKI which consistently lead to CKD. While patients with severe AKI can be kept alive with dialysis until renal recovery, this is not possible in mice and very severe AKI is fatal. This can be overcome by prolonged clamping of one kidney, inducing a very severe injury, and then removing the healthy kidney after the injured kidney has recovered. If the injured kidney has not recovered sufficiently before CN there is potential for animal suffering. To address this, we used MSOT to establish the time required for sufficient recovery of function. We first performed a pilot experiment with 40 min unilateral clamping, and monitored single kidney function in both the healthy and injured kidney with MSOT over 4 weeks by longitudinally assessing the pharmacokinetics (PK) of IRDye in the renal cortex (Figure S2).

**FIGURE 5:**
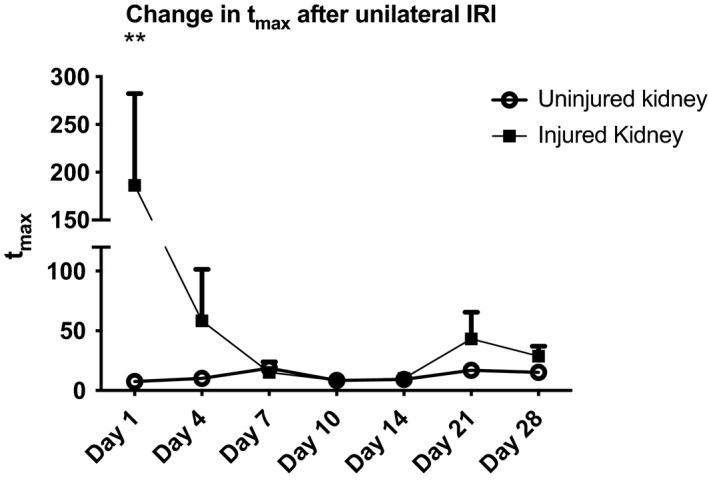
MSOT analysis of renal function in the CKD model. (a) Change in tmax of IRDye‐800 for the injured and uninjured kidney after IRI. On day 1 after injury there is a significant difference in function between the kidneys and an impression of the decline in function after day 14

We established the most appropriate model for monitoring IRDye PK by testing the fit of bi‐exponential and tri‐exponential models to MSOT curves. Tri‐exponential modelling fitted better than other models, both visually (Figure S5) and using the Akaike Information Criterion (Kingdom & Prins, 2016). The *t_max_* (time between administration of IRDye and maximum peak of the curve) distinguished between injured and uninjured kidneys consistently and was therefore further tested using linear mixed effects models. Using the uninjured kidney as a reference group there were significant differences between injured and uninjured kidney (*p *= 0.044) on day 1 (Figure 5). The injured kidney's function improved to levels equivalent to the uninjured kidney in week 2 and then declined significantly over the subsequent 2 weeks.

**FIGURE 6:**
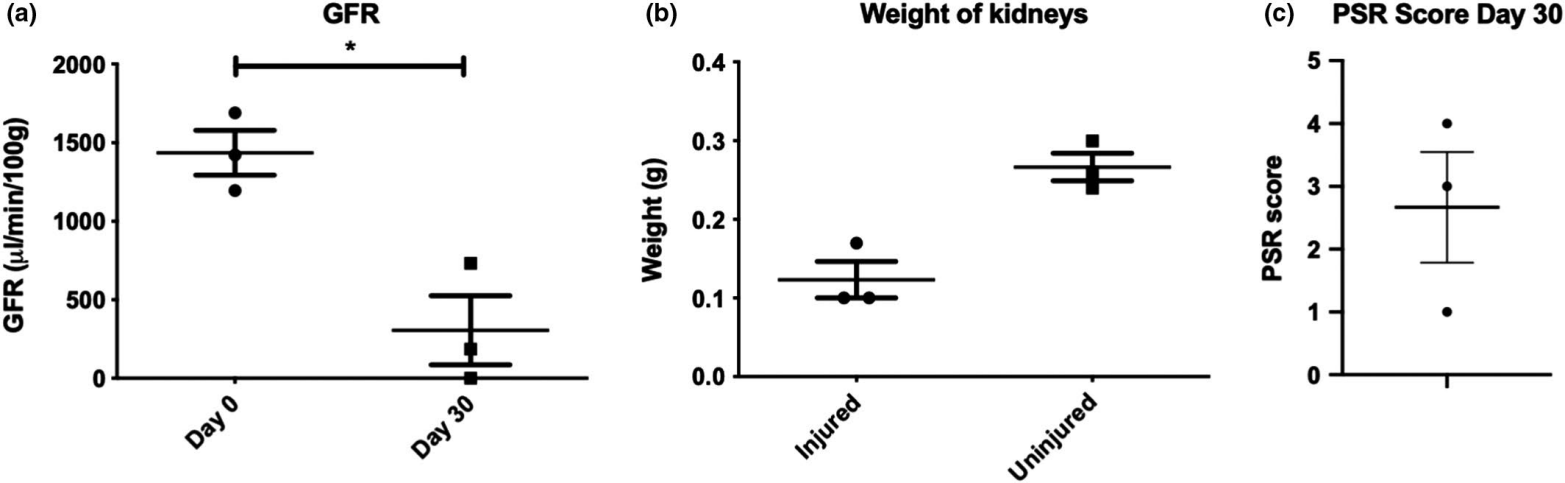
Renal injury in the clamped kidney after nephrectomy. (a) GFR prior to IRI injury vs after R‐IRI and contralateral nephrectomy shows significant reduction in GFR (Day 0: 1437 μl/min/100 g ± 248; Day 30: 307 μl/min/100 g ± 381, *p* 0.02). (b) The injured kidney weighs less than the uninjured kidney, showing loss of renal mass after prolonged R‐IRI (Injured: 0.12g ± 0.04, Uninjured: 0.27 g ± 0.03, *p* 0.07). (c) All injured kidneys displayed histological evidence of collagen deposition when stained with Picrosirius Red (PSR). No collagen deposition is seen in uninjured kidneys

GFR measurements performed at the experimental endpoint revealed significant reduction in GFR to an average of 20% of baseline function (Figure 6a), showing progression from AKI to CKD. Serum markers of function reflected significant injury above published normal ranges (Table S2) (River, 2012; Song et al., 2009). All injured kidneys weighed less than uninjured kidneys, and there was collagen deposition within all injured kidneys (Figure 6b,c). The impaired GFR at the study endpoint, accompanied with increased levels of serum biomarkers and histological damage, indicated that 40 min clamping with subsequent nephrectomy produces a severe renal injury resulting in CKD.

### 3.4 Refinement of a unilateral model of R‐IRI‐induced CKD results in a survivable, reproducible, and persistent renal injury

In the pilot study of unilateral R‐IRI and subsequent CN, MSOT showed a nadir of injury between days 10 and 14 (Figure 5a). Performing CN when the injured kidney is functioning optimally reduces suffering and improves survival. It also enables transdermal FITC‐Sinistrin clearance to be measured, which has the advantage that it can be undertaken in awake animals (Huang & Gretz, 2017; Scarfe et al., 2015). Therefore, the CKD model was refined by performing CN on day 14 after R‐IRI (Figure S2b).

**FIGURE 7:**
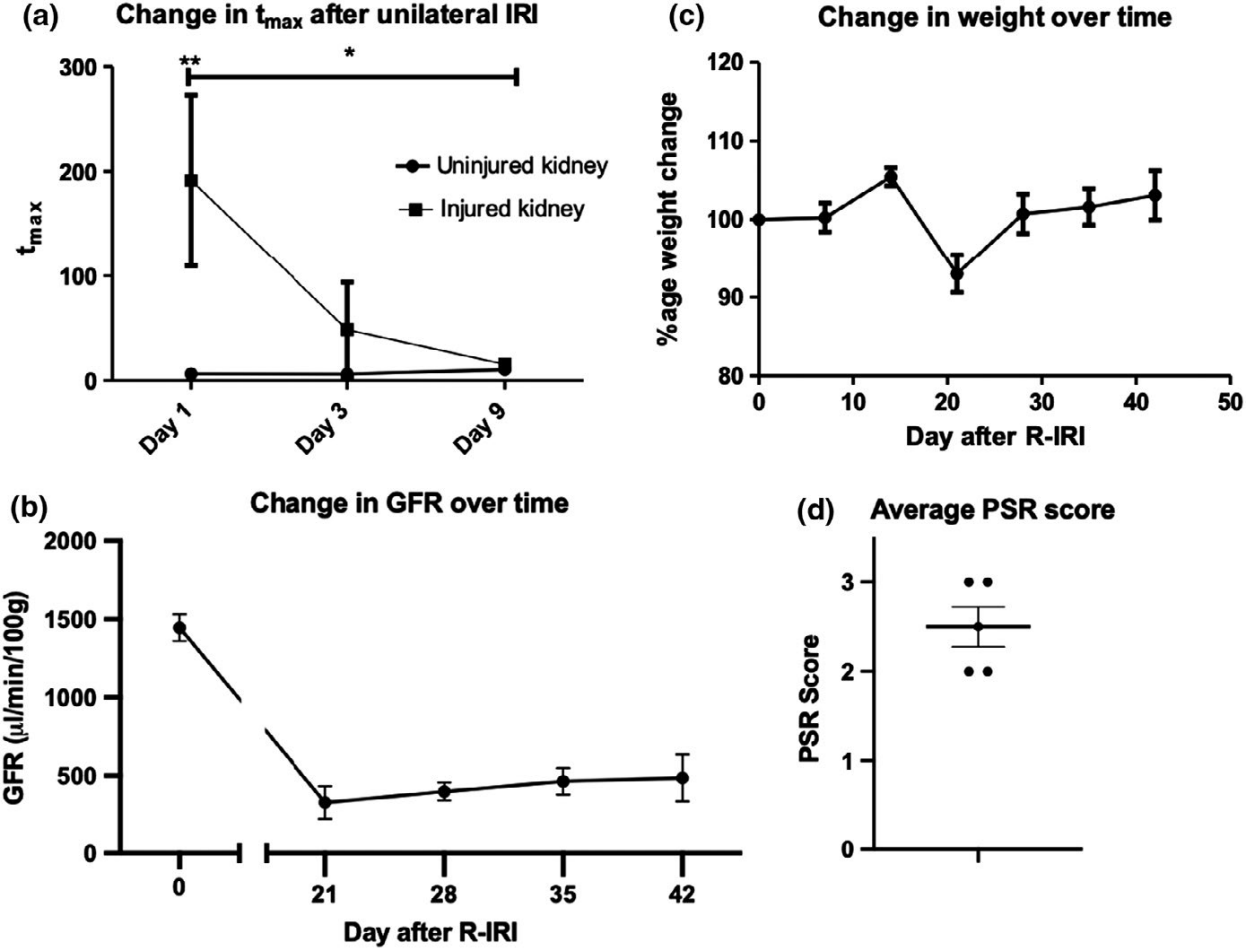
Refined CKD model. (a) The tmax differentiates between the injured and uninjured kidney on day 1 (*p* 0.002). There is significant reduction in tmax after injury (*p* 0.04), showing recovery of the kidney. *p < 0.05, **p < 0.01. (b) There is a persistent and significant reduction in GFR after contralateral nephrectomy. (c) There is no significant change in weight. (d) All animals had consistent levels of collagen deposition after staining with PSR. *n* = 6 in A; *n* = 5 in BD

In the refined study, the t_max_ differentiated between the injured and uninjured kidney on day 1 after R‐IRI and significant improvement in t_max_ was seen between day 1 and day 9 after R‐IRI (Figure 7a). One animal was excluded after developing diarrhea on day 12. Following CN, all animals had significant, persistent reduction in GFR (Figure 7b), without any significant weight change (Figure 7c). All animals had elevated serum markers of kidney injury (Table S1) and consistent collagen deposition (Figure 7d), demonstrating that MSOT is suitable for monitoring individual kidney function after R‐IRI using the t_max_ of IRDye clearance, and that doing so results in a tightly controlled model of AKI to CKD progression.

Overall, we show that performing a CN on day 14 after R‐IRI caused consistent, survivable CKD which can be monitored longitudinally with transdermal FITC‐Sinistrin clearance.

## 4 DISCUSSION

Here we report, for the first time, how noninvasive monitoring of renal function can be used to identify factors which add variability to the R‐IRI model, leading to its refinement. R‐IRI represents a variety of clinical situations (Ortiz et al., 2015) and its applicability to clinical practice is becoming increasingly evident with the recognition that one episode of ischemic AKI can lead to nephron loss, tubule‐interstitial atrophy, and fibrosis.

Most clinical causes of R‐IRI AKI affect both kidneys, therefore a bilateral R‐IRI model is appropriate for AKI studies. Clinically, a severe episode of AKI is associated with a risk of developing CKD (Hsu & Hsu, 2016) and the finding of collagen deposition 14 days after R‐IRI AKI supports this.

Description of the GFR as both an absolute and proportional measure of the baseline GFR enables the RIFLE classification (Bellomo et al., 2004) to be applied to loss of renal function. This approach to describing the GFR is novel and enables results to be reported using clinically relevant descriptors and enables appropriate power calculations to be performed for therapeutic studies.

When considering CKD a survivable but severe, consistent model of kidney injury was required. Prolonged unilateral clamping with delayed nephrectomy caused a consistent, reproducible reduction in GFR with more collagen deposition, and less variability while reducing animal suffering.

### 4.1 Standardization of pre‐clamping anesthetic time achieves a more reproducible R‐IRI model

Refinement of the R‐IRI model of AKI was possible because the duration of anesthetic prior to surgery has a significant impact on day 1 GFR. Older studies have suggested that isoflurane is renoprotective (Kim et al., 2013) but recent work disputes these findings (Gibbs et al., 2019) and we find that isoflurane did not prevent severe injury after R‐IRI. We postulate that the duration of isoflurane anesthetic is important due to its hypotensive effects (Constantinides et al., 2011). While invasive hemodynamic monitoring within the experiments may provide additional insight into this, the additional stress which this would cause to the animal precluded its use in these studies. Instead, standardizing this variable enables prioritization of tasks during R‐IRI surgery to achieve a more reproducible model. Standardization causes a reduction in both GFR and variability, giving the potential to reduce the number of animals required to show therapeutic efficacy.

### 4.2 MSOT can be used to determine the function of an injured kidney after R‐IRI

Previous publications have described measuring the delay between t_max_ in the renal cortex and pelvis (Scarfe et al., 2015) using MSOT, and the area under the curve (AUC) after drug‐induced global renal injury (Sharkey et al., 2019). Neither of these could be applied to the unilateral R‐IRI model due to the difficulty of imaging both renal pelvises simultaneously. This problem is compounded following surgery due to postoperative edema and over‐lying sutures. However, it was possible to accurately identify and image both renal cortices simultaneously after R‐IRI, and obtain consistent t_max_ measurements.

While it is not appropriate to use IRDye 800 clearance to calculate GFR, due to its high levels of protein binding (Huang & Gretz, 2017; Scarfe et al., 2015), it can be used as a surrogate measure of renal function due to its sole excretion through glomeruli. After assessment of IRDye 800 PK, we found that t_max_ is the parameter that best distinguishes the injured and uninjured kidney. We determined that the injured kidney recovered by day 10 with subsequent prolongation of t_max_, indicating the progression from functional AKI to CKD. While the development of CKD after AKI has been well described after R‐IRI (Zager, 2014), this is the first time that MSOT has been used to track CKD development longitudinally.

### 4.3 Contralateral nephrectomy on day 14 after prolonged unilateral R‐IRI produced a survivable, reproducible CKD model

Refinement of the CKD model was undertaken by performing CN on day 14. While reduction in GFR persisted after CN, a slight nonsignificant upward trend was seen which probably represented the adaptation that has been described due to glomerular hyperfiltration (Kwon et al., 2017). However, the week 6 GFR remained significantly reduced compared to baseline levels. Development of this model demonstrates the utility of long‐term accurate GFR monitoring, particularly when assessing the impact of therapeutic agents in an animal with predictable CKD.

In conclusion, in vivo noninvasive measures of renal function have enabled the refinement of AKI and CKD models after R‐IRI. Transdermal GFR measurements correlate well with histological markers of kidney disease and enable the refinement and reduction of animals required for future studies of therapies in these models. MSOT is feasible in animals following R‐IRI surgery and modelling of the clearance of IRDye using a tri‐exponential curve and t_max_ enables reproducible monitoring of the function of the injured and uninjured kidney.

Application of the RIFLE criteria for GFR to preclinical studies enables the translation of results to the clinic, ensuring that therapeutic studies show clinically important differences.

## CONFLICT OF INTEREST

No authors have any conflict of interest to report. The results presented in this paper are available in bioRxiv but have not been otherwise published.

## AUTHOR CONTRIBUTIONS

Funding for this work was secured by PM, BW, SK, PC, AO, and RH. RH, PM, BW, SK, PC, and AO designed the experiments. RH, LS, and JS performed the animal studies including surgical intervention, measurements of FITC‐Sinistrin, and IRDye 800 clearance and analysis of weight change, serum creatinine, and urea and Cystatin C. LR and RH performed the histological analysis. JB and GC performed the MSOT data analysis, RH, JB, and GC undertook the statistical analysis. RH, JB, PM, BW, and PC wrote the manuscript and generated the figures and all authors reviewed the manuscript prior to publication.

## DATA AVAILABILITY STATEMENT

FAll data are incorporated into the article and its online supplementary information. Raw data values are available upon request.

## Supporting information



Supplementary Material
